# ERG function in prostate cells is regulated by distinct positive feedback loops depending on AKT signaling status

**DOI:** 10.21203/rs.3.rs-9941989/v1

**Published:** 2026-06-15

**Authors:** Peter Hollenhorst, Saranya Rajendran, Ethan Golditch, Renee Kinne, Nicholas Downing, Kenneth P. Nephew, Sean McCabe

**Affiliations:** Indiana University School of Medicine; Indiana University Melvin and Bren Simon Comprehensive Cancer Center

## Abstract

A chromosomal rearrangement that causes aberrant expression of the ERG transcription factor is the most common genomic alteration in prostate cancer. Expression of ERG in prostate cells can promote either luminal epithelial or mesenchymal cell fates depending on the status of the PI3K/AKT signaling pathway. Phosphorylation of ERG by ERK is important for oncogenic activity and can be regulated by the TLR4 signaling pathway. Here we found that the PI3K/AKT pathway not only regulated how ERG promoted distinct cell types within prostate cells, but also altered the positive-feedback loops that drove ERG phosphorylation. Activation of AKT signaling switched the upstream pathway that promotes ERG phosphorylation from TLR4 to VEGF. Inhibition of the PI3K/AKT pathway in ERG-positive prostate cells resulted in loss of androgen receptor expression, gain of mesenchymal markers and loss of sensitivity to androgen receptor inhibition. Further, androgen depletion reduced PI3K/AKT activity while increasing MEK/ERK activity and relative ERG phosphorylation. In a xenograft model, single agent inhibition of TLR4 or VEGFA reduced ERG phosphorylation, tumor size, and luminal marker expression but increased mesenchymal marker expression. Combination TLR4/VEGFA inhibition reduced tumor size and reduced both luminal and mesenchymal markers. These findings suggest novel combinatorial treatments for ERG-positive prostate cancer.

## BACKGROUND

The most common oncogene in prostate cancer is the gene encoding the ETS family transcription factor ERG [1]. Chromosomal rearrangements resulting in ERG expression occur in up to 50% of prostate tumors [2]. ERG is not normally expressed in prostate epithelial cells, and mouse models indicate that ERG expression in prostate cells, coupled with second-hit mutations, such as those that can activate PI3K/AKT signaling, can drive prostate tumorigenesis [3–5]. Despite the central importance of ERG in a large fraction of prostate tumors, there are currently no precision therapeutic strategies for ERG-positive disease.

Aberrant ERG expression in prostate epithelial cells can promote changes in cell fate. Expression of ERG in the immortalized-normal prostate epithelial cell line RWPE1, drives cell migration and expression of markers associated with epithelial to mesenchymal transition (EMT) [6–8]. In contrast, studies using genetically engineered mice have found that ERG can promote luminal epithelial differentiation [9, 10]. The difference in these cell fate decisions appears to be due to PI3K/AKT signaling status, as expression of constitutively active AKT with ERG in RWPE1 cells results in a loss of EMT markers, and a gain of luminal epithelial markers [11]. Prior studies that found ERG promotes luminal differentiation also include mutations that activate PI3K/AKT signaling such as *PTEN* deletion [9]. AKT activation altered the cistrome of ERG [11], suggesting that ERG can drive different cell fates by regulating different sets of target genes dependent on AKT signaling status.

ERG phosphorylation by ERK is necessary for ERG to drive tumorigenesis and other phenotypes such as cell migration and clonogenic growth [11, 12]. ERK first phosphorylates ERG at S215 which alters the conformation of ERG allowing ERK to phosphorylate ERG S96 [12]. ERG S96 phosphorylation releases a co-repressor complex consisting of FOXO1 and polycomb repressive complex 2 (PRC2) allowing ERG to activate transcription [11, 13]. When ERG is expressed in RWPE1 prostate cells, S96 phosphorylation is driven by a positive feedback loop with the TLR4 signaling pathway [14]. In these cells ERG expression drives TLR4 expression, which results in activation of ERK, which phosphorylates ERG. Further, inhibition of TLR4 by the small molecule inhibitor TAK-242 (Resatorvid) can inhibit ERG function in RWPE1 cells [14].

Expression of ERG along with constitutively active AKT (myristoylated AKT: mAKT) in RWPE1 cells drives clonogenic growth in vitro, and tumorigenesis in a xenograft model [11]. These RWPE-ERG-mAKT tumors express luminal epithelial markers including androgen receptor (AR). When the phosphomimetic mutant ERG S96E is expressed in RWPE1 cells, it can drive clonogenic growth and tumorigenesis as a single-hit, without AKT activation [11]. RWPE-ERG S96E tumors lack luminal markers, and express basal epithelial markers such as p63 and KRT5. Thus, ERG can promote tumorigenesis with or without luminal epithelial cell fates.

In this study we find that expression of ERG with mAKT decreases the number of basal epithelial and mesenchymal cells in culture and increases the number of luminal epithelial and luminal progenitor cells. Further, when mAKT is present, the signaling pathway that regulates ERG S96 phosphorylation switches from TLR4 to VEGF. Inhibition of VEGFA by bevacizumab reduced ERG S96 phosphorylation and reduced ERG function in cells with mAKT. Inhibition of AKT in ERG-positive VCaP prostate cancer cells reduced luminal epithelial markers and increased mesenchymal markers. Further, AKT inhibition eliminated the sensitivity of VCaP cells to the AR inhibitor enzalutamide. Inhibitors of both TLR4 and VEGFA reduced the growth of VCaP xenograft tumors. Further, the combined inhibition of TLR4 and VEGF reduced both mesenchymal and luminal markers. Together these findings suggest novel therapeutic combinations specific for ERG-positive prostate cancer.

## Materials and Methods

### Cell culture

Cell lines were authenticated within 1 year of use by a PowerPlex 16HS assay (Promega; Madison, WI), and were checked by PCR for mycoplasma contamination within five passages. RWPE1 cells were grown in keratinocyte serum-free medium (Sigma; Burlington, MA). VCaP and WPMY1 cells were maintained in Dulbecco’s modified Eagle’s medium (DMEM; Sigma) with 10% fetal bovine serum (FBS; Sigma). LNCaP and DU145 cells were maintained in RPMI medium (Sigma) with 10% FBS. All cells were incubated at 37 °C in 5% CO_2_ and all media included 1× penicillin/streptomycin (Sigma). Cells used in this study were passaged fewer than 20 times. A 10 mM stock solution of enzalutamide (S1250; SelleckChem; Houston, TX) was prepared in DMSO. A 10 mM stock solution of the TLR4 inhibitor TAK-242 (Fisher Scientific; Pittsburgh, PA), 10 mM stock solution of ipatasertib (MedChem Express; Monmouth Junction, NJ), and a 1 mM stock solution of LY294002 (Cell Signaling; Danvers, MA) were prepared in DMSO. Bevacizumab (HY-P9906) was from MedChem Express.

ERG and phospho-mutants were expressed in modified pLHCX where the CMV promoter is replaced with the *HNRNPA2B1* promoter as previously described [15]. pBabe-Puro-Myr-Flag-AKT1 was a gift from William Hahn (Addgene plasmid #15294) [16], and pBABE-puro was used as a vector control. HEK-293T cells were co-transfected with retroviral expression plasmids, PN8E gagpol delta5, and pN8E VSV-G plasmids to generate retroviral vectors. Supernatant was added to RWPE1 cells in the presence of 10 μg/ml polybrene for 4 hr, followed by replacement with growth medium. After 24 hr, cells were maintained under selection with hygromycin (250 μg/ml) and puromycin (20 μg/ml).

### Single-cell RNA-seq

10,000 cells were used as input to the 10X Genomics Chromium system using the Chromium Next GEM Single Cell 3' Kit v3.1. Libraries were sequenced at the IUSM Center for Medical Genomics in a NovaSeq 6000 with a NovaSeq S2 reagent kit. Cells were quantified using Cell Ranger 7.1.0. Genes with less than 5 cells with expression were removed. Quality control protocols for cells include requiring a minimum of 200 and a maximum of 2500 expressed genes per cell. Cells with a Mitochondrial percent less than 7.5 were also filtered out. The R package Harmony was used to integrate the two existing datasets after normalization and both UMAP and PCA dimension reduction was performed and used for visualization and clustering. A Wilcoxon Rank Sum test was performed per gene and marker genes were defined as having a Benjamini-Hochberg adjusted p-value less than 0.05. Raw and processed data files are available at Gene Expression Omnibus under accession number GSE330458.

### Co-culture assay

WPMY1 cells were labeled with a cytoplasmic green, fluorescent dye (BioTracker 490; Sigma), and RWPE1 cells were labeled with a cytoplasmic red fluorescent dye (BioTracker 655; Sigma). Labeled cells were mixed at a 1:1 ratio and co-cultured for 5 days in a medium consisting of keratinocyte serum-free medium and DMEM at a 1:1 ratio. Fluorescence images were acquired using a Nikon fluorescence microscope.

### Spheroid assay

WPMY-1 cells were co-cultured with RWPE1 cells in 24-well low-attachment plates (Sigma) containing a 1:1 mixture of keratinocyte serum-free medium and DMEM. For treatment conditions, cells were pretreated with TAK-242 (1 μM, 24 h) or bevacizumab (1 μM, 24 h), or left untreated, prior to plating. Culture medium was replaced every 3 days. Images were acquired on day 5 using a Zeiss Axiovert 40 inverted microscope. Spheroid area in each well was quantified using ImageJ software and graphs were generated using data from three independent biological replicates.

### Clonogenic survival assay

RWPE-ERG, RWPE-ERG-mAKT, VCaP, or DU145 cells were pretreated with TAK-242, bevacizumab, or a combination of both for 24 hr, or left untreated. Following pretreatment, cells were seeded at a density of 1,000 cells per well in 6-well culture plates (Sigma) containing pre-warmed growth medium. Plates were incubated for 7–10 days. Cells were fixed with 10% formalin and stained with 0.5% crystal violet in 25% methanol. Plates were air-dried, and colonies imaged and quantified using Genesys software (Syngene; Bangalore, India). Values are the mean of three biological replicates, each the mean of three technical replicates.

### Cell proliferation assay

VCaP cells were seeded in 96-well plates at a density of 1,000 cells per well and cultured for 24, 48, or 72 h. Cell proliferation was assessed using the MTT assay. Absorbance was measured at 490 nm using a microplate spectrophotometer.

Treatment conditions included VCaP cells cultured in complete medium, phenol-red free DMEM (VWR; Radnor, PA) with charcoal-stripped FBS (Sigma), and charcoal-stripped phenol-red free medium supplemented with R1881 (Sigma). Each condition was performed in triplicate biological replicates each including triplicate technical replicates.

### RNA isolation and quantitative RT–PCR

Total RNA was isolated from cells using the RNeasy Kit in combination with QIAshredder columns (Qiagen; Germantown, MD) according to the manufacturer’s instructions. β-mercaptoethanol (1%) was added to the RLT lysis buffer prior to extraction. RNA concentration and purity were determined using a NanoDrop 2000c spectrophotometer (Sigma).

Complementary DNA (cDNA) was synthesized by reverse transcription as previously described (24). Each reverse transcription reaction contained 1 μg total RNA, 500 μM dNTPs, 100 nM oligo primers, 1× First Strand Buffer (New England BioLabs; Ipswitch, MA), 5 mM dithiothreitol (DTT; Invitrogen; Carlsbad, CA), 40 U murine RNase inhibitor, and 200 U SuperScript III reverse transcriptase in a total reaction volume of 20 μl. Reactions were incubated at 55 °C for 55 min, followed by enzyme inactivation at 70 °C for 15 min. RNase H (5 U) was added and samples incubated at 37 °C for 20 min. Quantitative PCR (qPCR) reactions used 2 μl of cDNA, 2 μl RNase-free water, 1× KAPA SYBR Fast qPCR master mix (2.6 mM MgCl_2_), and 500 nM forward and reverse primers in a final reaction volume of 10 μl. Reactions were run on a QuantStudio system with the following cycling conditions: 95 °C for 3 min, followed by 40 cycles of 95 °C for 15 s, 60 °C for 15 s, and 72 °C for 30 s. Gene expression was normalized to 18S rRNA or GAPDH. Primer sequences are in Supplementary Table 1.

### Immunoprecipitation

Detection of ERG S96 phosphorylation required prior immunoprecipitation with a pan-ERG antibody. RWPE-1 cells were lysed in NP-40 lysis buffer, probe sonicated, and centrifuged. Clarified supernatant was incubated overnight at 4 °C with ERG antibody followed by anti-mouse Dynabeads for 2 h. Beads were washed four times with lysis buffer and bound proteins eluted in 4× Laemmli sample buffer.

### Immunoblotting

Whole-cell extracts were prepared using NP-40 lysis buffer (50 mM Tris–HCl, pH 7.4, 250 mM NaCl, 5 mM EDTA, 50 mM NaF, and 1% NP-40). Protein extracts were separated on 10% SDS-PAGE gels, transferred to nitrocellulose membranes (Bio-Rad; Hercules, CA), blocked in 5% milk in Tris-buffered saline(10 mM Tris, pH 8.0, 150 mM NaCl), incubated with primary antibodies followed by horseradish peroxidase (HRP)–conjugated secondary antibodies, and visualized using SuperSignal enhanced chemiluminescence (Sigma). Antibodies used in this study included ERG (CM421C, BioCare; Pacheco, CA), pERG S96 [12], pERG S215 [17], phospho-AKT (Cell Signaling; 4060), AKT (Cell Signaling; 4691), AR (Abcam; Waltham, MA; 108341), phospho-MEK (Cell Signaling; 9121), and GAPDH (Santa Cruz Biotechnology; Dallas, TX; 47724).

### Immunohistochemistry

Tumors excised from mice were fixed overnight in 10% formalin, embedded in paraffin, and sectioned. Sections were stained with hematoxylin and eosin (H&E) and imaged using a Leica light microscope at ×200 magnification. Immunohistochemical staining was performed using standard methods. Briefly, 5 μm sections were deparaffinized, endogenous peroxidase activity was quenched with 3% peroxide for 10 min, and antigen retrieval was performed in 0.1 M sodium citrate using a pressure cooker. Sections were blocked with 5% BSA and incubated overnight at 4 °C with polyclonal antibodies against fibronectin, KRT18 and pERG ^s215^. Detection used SignalStain^®^ Boost Detection Reagent (Cell Signaling; Rabbit; 8114) and SignalStain^®^ DAB Substrate Kit (Cell Signaling), followed by dehydration through graded alcohols and mounting. Slides were imaged using a Motic (San Francisco, CA) EasyScan scanner and analyzed with QuPath software.

### Mouse xenograft study

Animal studies were performed under IACUC protocol #23–011, and all animals were treated in accordance with NIH guidelines for laboratory animals and institutional animal care and use committee protocols at Indiana University, Bloomington. VCaP cells (3 × 10^6^) were mixed with Matrigel at a 1:1 ratio and injected subcutaneously into the right flank of male NSG mice. Tumor size was measured periodically using calipers, and tumor volume was calculated using the formula V = ½ × L × W^2^, where L represents the longest tumor diameter and W represents the perpendicular tumor diameter.

When tumors reached approximately 100 mm^3^, mice were randomized into four groups: vehicle control, TAK-242, bevacizumab, and combination treatment. Mice received intraperitoneal injections of vehicle (DMSO), TAK-242 (20 mg/kg), bevacizumab (5 mg/kg), or both twice weekly for 3 weeks. Dosage and treatment regimen were guided by a previously published TAK-242 mouse study [18]. Tumor size and mouse body weight were monitored throughout the study. At the conclusion of the experiment, mice were euthanized and tumors were harvested for subsequent analyses.

## RESULTS

### ERG can promote distinct cell fates.

**Figure 1:**
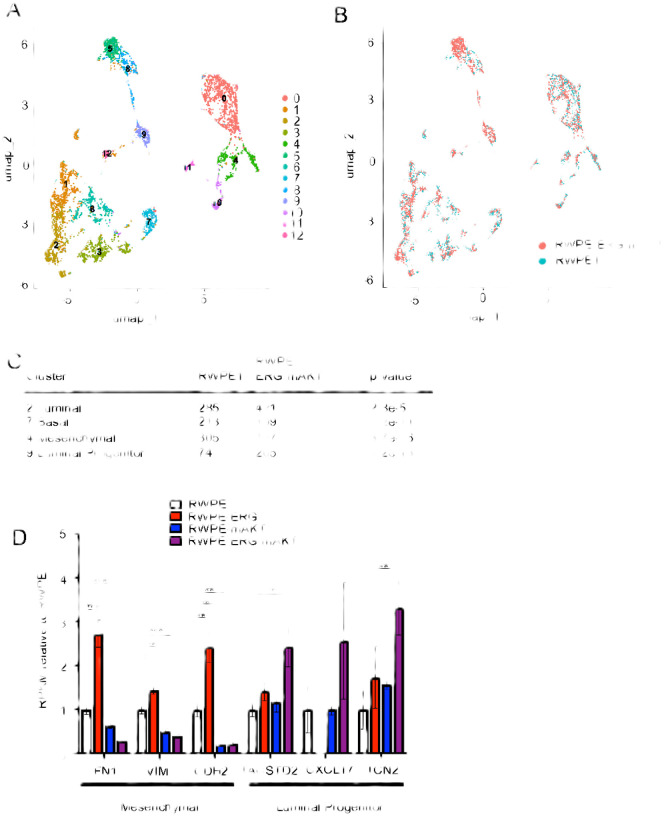
ERG promotes distinct cell types (**A**) Uniform Manifold Approximation and Projection (UMAP) dot plot of combined RWPE1 and RWPEERG-mAKT cell lines colored by cluster. (**B**) Dot plot as in (A), except colored by RWPE1 (blue) or RWPE-ERG-mAKT (red) (**C**) Number of cells in the indicated clusters in each cell type (**D**) Reads per kilobase per million (RPKM) for the indicated mesenchymal or luminal progenitor marker genes in the indicated cell lines from a published RNA-seq dataset (11). Statistical significance was determined using a one-way ANOVA test. Data are presented as mean ± SD (standard deviation) with n = 3. *p < 0.05, **p < 0.01, ***p < 0.001.

Expression of ERG in the immortalized-normal prostate cell line RWPE1 drives both mesenchymal and basal epithelial marker gene expression [11]. However, expression of ERG in a background of constitutively active AKT (myristoylated AKT: mAKT) promotes loss of mesenchymal and basal epithelial markers and gain of luminal epithelial markers [11]. It is unclear if these changes represent relative gain or loss of distinct cell populations, or changes in marker gene expression in a single homogenous population of cells. To test this, single-cell RNA sequencing was used to analyze both parental RWPE1 cells and RWPE1 cells expressing ERG and mAKT (RWPE-ERG-mAKT). Consistent with previous single-cell analysis of clonal cell lines grown in culture [19], distinct cell populations were identified (Fig. 1a). 13 total clusters were defined. To identify clusters representing luminal and basal epithelial cells, luminal (*KRT18*, *KRT8*) and basal (*KRT5*, *TP63*) markers were examined. Cluster 2 showed a small, but significant enrichment of luminal epithelial markers (Supplementary Table 2). Cluster 7 similarly showed a small but significant enrichment of basal epithelial markers (Supplementary Table 2). In contrast to these relatively small enrichments, cluster 4 displayed larger enrichment for mesenchymal markers Vimentin (*VIM*), N-cadherin (*CDH2*), and fibronectin (*FN1*) (Supplementary Table 2). In addition, a novel cell population was identified in cluster 9 with significant enrichment for prostate luminal progenitor markers *TACSTD2* [20] and *CXCL17* [21], and the kallikrein genes *KLK10*, *KLK8*, and *KLK5* (Table S2). Upon expression of ERG and mAKT in RWPE1 cells, a shift in cell populations was observed with significant loss of basal epithelial and mesenchymal cell clusters and significant gain of luminal epithelial and putative luminal progenitor cell clusters (Fig. 1b and c). To test if this function was ERG-dependent, rather than an effect of mAKT alone, we analyzed mesenchymal and luminal progenitor markers within previously published bulk RNA-seq of RWPE1, RWPE-ERG, RWPE-mAKT and RWPE-ERG-mAKT. Mesenchymal markers were upregulated in RWPE-ERG, but down-regulated in RWPE-mAKT and RWPE-ERG-mAKT (Fig. 1d). Luminal progenitor markers were up-regulated in RWPE-ERG-mAKT compared to all other groups, including RWPE-mAKT. Therefore, ERG can promote changes in the relative proportion of distinct cell populations including a gain in luminal progenitor cells in the presence of activated AKT.

### ERG promotes sphere-formation regardless of cell fate.

**Figure 2:**
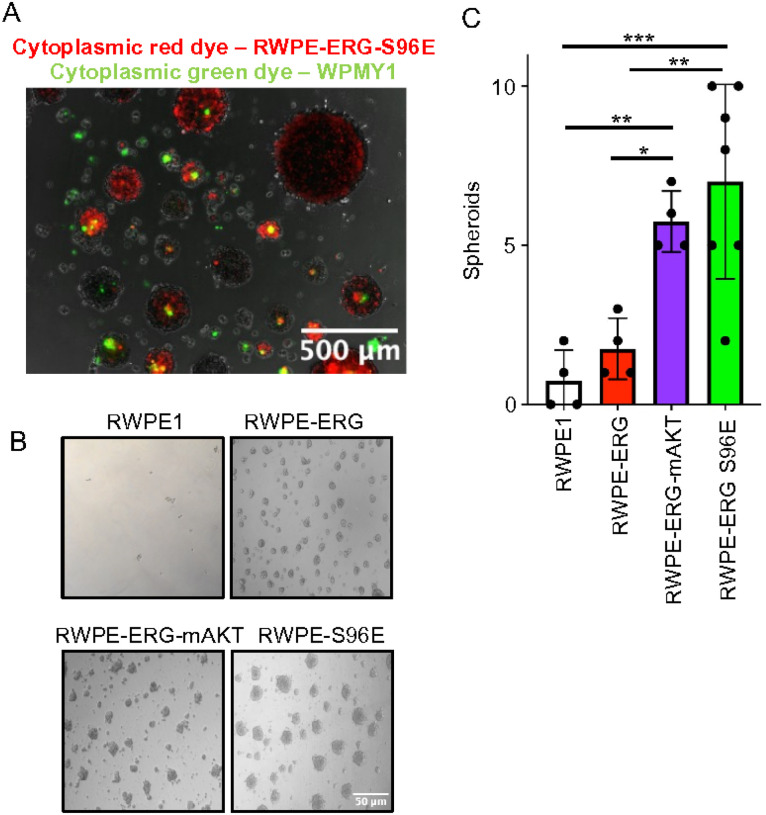
ERG promotes sphere formation regardless of cell fate. (**A**) Image of prostate cell co-culture. Epithelial RWPE-ERG-S96E cells (red) and stromal WPMY1 cells (green) were co-cultured for 5 days, during which prostaspheres formed. (**B**) Representative images of WPMY1 co-culture with indicated RWPE1 derived constructs (**C**) Graphs show the number of spheroids formed by RWPE1, RWPE-ERG, RWPE-ERG-mAKT, and RWPE-ERG S96E cells on day 9 of culture. The diameter of cell aggregates was calculated from bright-field images using ImageJ software. Cell aggregates with a diameter >35 μm were classified as spheroids. Statistical significance was determined using a one-way ANOVA test. Data are presented as mean ± SD *p < 0.05, ***p < 0.001.

A possible role for ERG in promoting a luminal progenitor cell fate indicates that ERG might promote stemness attributes. To test a phenotype associated with stemness, spheroid formation was assessed. It was previously shown that RWPE1 prostate epithelial cells co-cultured with WPMY1 prostate stromal cells will form organoid-like spheroid structures [22]. Spheroid formation was observed using this co-culture system (Fig. 2a). RWPE1 cells were incubated with a red cytoplasmic stain and WPMY1 cells with a green cytoplasmic stain prior to co-culture. Spheres had a core of green stromal cells and an outer layer of red epithelial cells (Fig. 2a), consistent with the previous report [22]. The addition of ERG alone to the RWPE1 cells resulted in a small increase in sphere formation (Fig. 2b and c). However, expression of ERG with mAKT or expression of the phosphomimetic ERG mutant S96E resulted in a significant increase in the number of large spheres compared to wild-type ERG alone (Fig. 2b and c). This result correlates with findings from previously reported xenograft tumor formation assays, where RWPE-ERG-mAKT and RWPE-ERG S96E form significantly larger tumors than RWPE-ERG [11]. These findings suggest that ERG can promote stemness related phenotypes that correlate with a tumor formation function. However, this stemness function is distinct from the effect of ERG on cell fate, as RWPE-ERG-mAKT and RWPE-S96E promote very distinct cell states (luminal and luminal progenitor versus basal and mesenchymal, respectively).

### Multiple signaling pathways can promote ERG phosphorylation and function.

**Figure 3:**
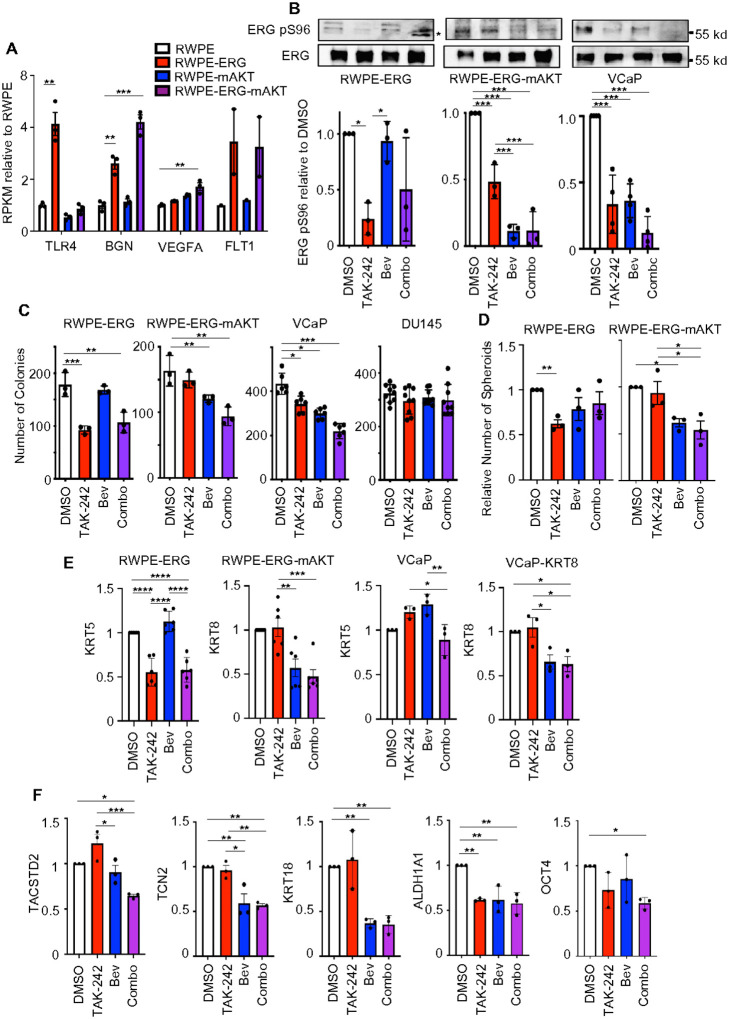
Signaling pathways that promote ERG phosphorylation can change. (**A**) Reads per kilobase per million (RPKM) for *TLR4*, *BGN*, *VEGFA*, and *FLT1* in the indicated cell lines from a published RNA-seq dataset. (**B**) Western blot analysis of lysates prepared from RWPE-ERG, RWPE-ERG-mAKT, and VCaP cells treated with DMSO, TAK-242 (1 μM), bevacizumab (1 μM), or the combination for 24 hr. Graphs show mean densitometric analysis ± SEM for pERG S96 (N = 3), normalized to total ERG and shown relative to DMSO (**C**) The number of colonies in RWPE-ERG, RWPE-ERG-mAKT, VCaP, and DU-145 cells pretreated with DMSO, TAK-242 (1 μM), bevacizumab (1 μM), or the combination for 24 hr was quantified (**D**) Relative number of spheroids formed by RWPE-ERG and RWPE-ERG-mAKT cells pretreated with DMSO, TAK-242 (1 μM), bevacizumab (1 μM), or the combination for 24 hours and analyzed on day 9 of culture (**E**) Expression of *KRT5* or *KRT8* in indicated cell line treated as indicated for 24 hr measured by qRT-PCR and shown relative to 18s rRNA, then relative to DMSO. (**F**) Expression of indicated genes by qRT-PCR (as in E) of RNA isolated from spheroids of WPMY1 plus RWPE-ERG-mAKT cells treated with vehicle control, TAK-242, bevacizumab, or the combination for 24 hr. All significance determined by one-way ANOVA with *p ≤ 0.05, **p < 0.01, ***p ≤ 0.001.

Phosphorylation of ERG at S96 is critical for ERG to promote tumor formation [11]. We previously found that the TLR4 pathway functions in a feed-forward loop with ERG in that ERG activates TLR4 expression, which increases ERK activity, which phosphorylates and activates ERG [14]. Analysis of published RNA-seq data [11] confirms that ERG promotes expression of *TLR4* and the non-canonical TLR4 ligand *BGN* (Fig. 3a). However, *TLR4* was no longer activated by ERG in the presence of mAKT (Fig. 3a), indicating that this feed-forward loop may not be functioning in backgrounds with high AKT activity. We previously found that mAKT alters the cistrome of ERG [11]. The most over-represented ontology for new ERG target genes in the presence of mAKT was “blood vessel formation”. A study by Fish et al. found that VEGF signaling regulates ERG phosphorylation in blood vessels [23]. RNA-seq indicates that VEGFA and the VEGFA receptor FLT1 are activated by ERG in the presence of mAKT (Fig. 3a).

To test roles of TLR4 and VEGFA signaling in ERG regulation we compared ERG phosphorylation after treatment with the TLR4 inhibitor TAK-242 or the VEGFA signaling inhibitor bevacizumab. In RWPE-ERG cells, TAK-242 significantly decreased ERG S96 phosphorylation but bevacizumab had no effect (Fig. 3b). The combination treatment consistently resulted in a higher mobility band (Asterisk, Fig. 3b) of unknown origin only in RWPE-ERG cells and made quantification of this data point difficult. In contrast to RWPE-ERG cells, bevacizumab treatment decreased ERG S96 phosphorylation in RWPE-ERG-mAKT cells (Fig. 3b), suggesting that the VEGFA pathway can promote ERG S96 phosphorylation when AKT is active. In VCaP prostate cancer cells, which have *TMPRSS2::ERG* rearrangement, both TAK-242 and bevacizumab decreased ERG phosphorylation (Fig. 3b). Therefore, both TLR4 and VEGFA signaling can regulate ERG phosphorylation.

To test the effect of inhibiting these pathways on ERG function, we examined two phenotypes promoted by ERG, clonogenic growth and spheroid formation. Similar to the S96 phosphorylation results, TAK-242, but not bevacizumab, reduced colony formation in RWPE-ERG cells, while bevacizumab decreased colony formation in RWPE-ERG-mAKT cells. In VCaP cells both treatments reduced colony formation and the combination was most effective (Fig. 3c). As a control, DU145 prostate cancer cells, which do not express ERG, were tested and TAK-242 and bevacizumab did not change clonogenic growth (Fig. 3c).

Results in the RWPE/WPMY co-culture spheroid assay were similar in that TAK-242 reduced spheroid formation in RWPE-ERG cells only, and bevacizumab reduced spheroid formation in RWPE-ERG-mAKT cells only (Fig. 3d).

We then tested the role of these inhibitors on the expression of marker genes of the cell lineages promoted by ERG (Fig. 3e). In RWPE-ERG cells, where ERG promotes mesenchymal and basal epithelial gene expression, TAK-242, but not bevacizumab, decreased expression of the basal epithelial marker gene *KRT5*. In RWPE-ERG-mAKT cells, where ERG promotes luminal epithelial marker expression, bevacizumab, but not TAK-242, decreased expression of the luminal epithelial marker *KRT8*. In VCaP cells, which express androgen receptor and other luminal markers, neither inhibitor altered expression of KRT5, but bevacizumab reduced expression of *KRT8*.

Finally, to test roles of the inhibitors in regulation of stemness related genes, RWPE-ERG-mAKT cells grown as spheroids in co-culture with WPMY1 cells were treated and gene expression measured (Fig. 3f). The luminal progenitor marker *TACSTD2* and the stemness marker *OCT4* were reduced in the combination treatment. The luminal progenitor marker *TCN2* and the luminal marker KRT18 were reduced by single agent bevacizumab treatment. The stemness marker *ALDH1A1* was reduced by both TAK-242 and bevacizumab as single agents. Based on these findings, both TLR4 and VEGF signaling can promote the expression of stemness markers.

### Inhibiting AKT results in loss of luminal markers and promotes EMT in ERG-positive prostate cancer cells.

**Figure 4:**
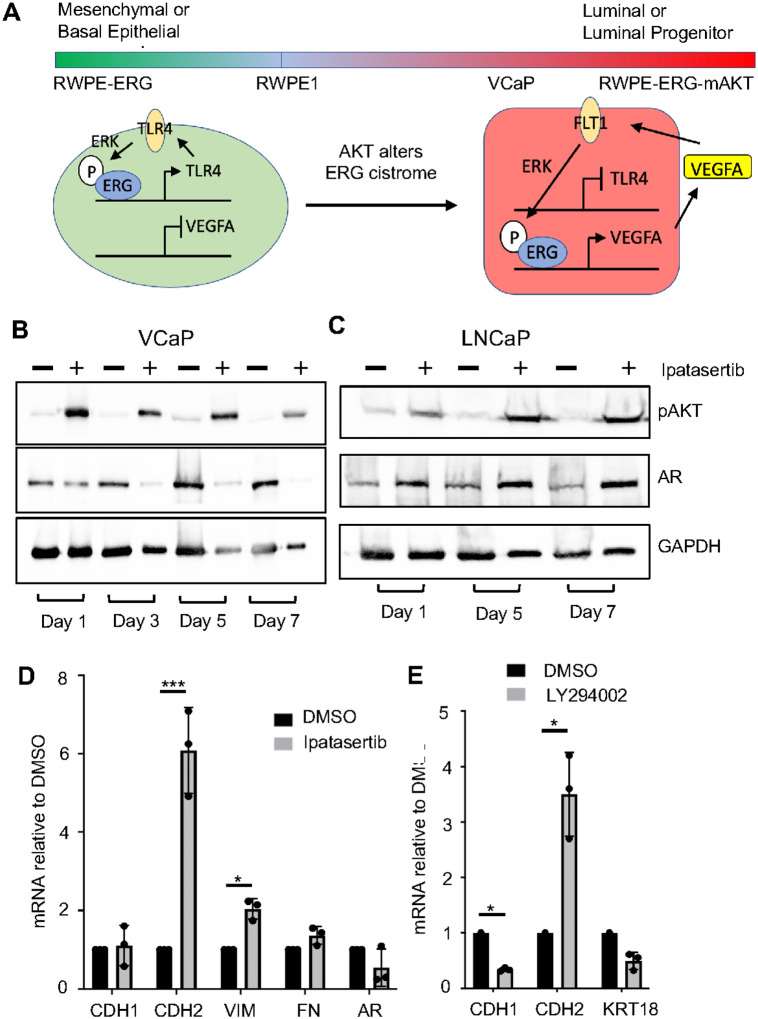
Inhibiting AKT results in loss of luminal markers and EMT in ERG-positive prostate cancer cells. (**A**) Model illustrating how ERG may be differentially regulated in distinct cell types. Positive feedback loops involving either the VEGF pathway or the TLR4 pathway may function to stabilize specific differentiated states. (**B**) Immunoblot analysis of lysates prepared from VCaP cells treated with DMSO or ipatasertib (1 μM) for 1, 3, 5, and 7 days. (**C**) Immunoblot analysis of lysates prepared from LNCaP cells treated with DMSO or ipatasertib (1 μM) for 1, 5, and 7 days. (**D**) Expression by qRT-PCR of indicated genes in VCaP cells treated with DMSO or ipatasertib for 5 days. Levels shown relative to 18s rRNA, then relative to DMSO control. (**E**) Expression of *CDH1*, *CDH2*, and *KRT18* in VCaP cells treated with DMSO or LY294002 for 5 days measured as in D. All significance was determined by one-way ANOVA with *p ≤ 0.05, ***p ≤ 0.001.

Our findings suggest a model where ERG activity promotes mesenchymal and basal epithelial cell fates in a positive feedback loop with the TLR4 pathway when AKT signaling is low and promotes luminal and luminal progenitor cell fates in a positive feedback loop with the VEGFA pathway when AKT signaling is high (Fig. 4a). This model predicts that AKT inhibition in an ERG-positive prostate cancer cell would decrease luminal markers and increase mesenchymal or basal epithelial markers. To test this, ERG-positive and negative cell lines were treated with the AKT inhibitor ipatasertib. Consistent with previous findings [24], AKT-inhibition by ipatasertib increased phospho-AKT levels (Fig. 4b and c). Treatment of ERG-positive VCaP cells with ipatasertib resulted in a time-dependent decrease in the luminal marker androgen receptor (AR) (Fig. 4b). In contrast, AR levels in ERG-negative LNCaP cells increased with ipatasertib treatment (Fig. 4c). Expression of additional marker genes were measured by RT-qPCR. Mesenchymal markers N-cadherin (*CDH2*) and vimentin (*VIM*) increased when VCaP cells were treated with ipatasertib (Fig. 4d). The PI3K inhibitor LY294002, which inhibits upstream activation of AKT was also tested. PI3K inhibition of VCaP cells decreased expression of the epithelial marker E-cadherin (*CDH1*) and increased expression of the mesenchymal marker N-cadherin (Fig. 4e), suggesting an epithelial to mesenchymal transition (EMT). Together these data indicate that repression of AKT leads to loss of luminal character and EMT in ERG-positive prostate cancer cells specifically.

### Decreased AKT signaling reduces AR dependence in ERG-positive VCaP cells

**Figure 5:**
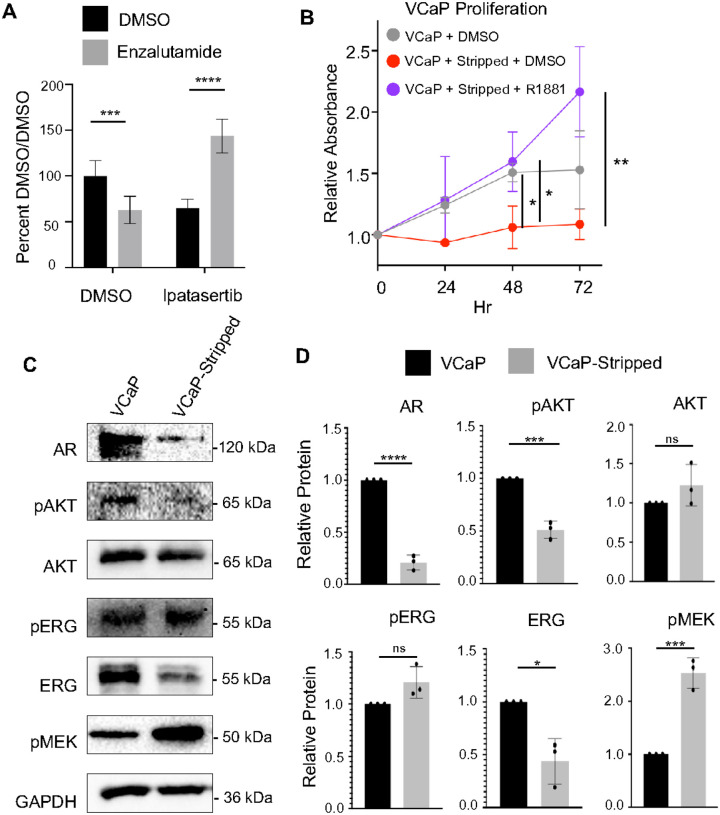
VCaP cells shift from PI3K/AKT to Ras/MAPK signaling upon androgen deprivation. (**A**) Viable cell number relative to DMSO/DMSO by MTT 7 days after 10 mM enzalutamide or DMSO, following 3-day pre-treatment with 5 mM ipatasertib or DMSO (n=12). (**B**) VCaP cells were seeded at 20,000 cells per well in a 96-well plate in complete medium, charcoal-stripped DMEM, or charcoal-stripped DMEM with 10 nM R1881. Relative cell number was measured using an MTT assay with three biological replicates, each the mean of three technical replicates. (**C**) Representative images of immunoblots of VCaP cell lysates grown in either complete or charcoal-stripped DMEM for 5 days. (**D**) Densitometric analysis of immunoblots (n=3). Data were normalized to *GAPDH* signal and to VCaP cells grown in complete medium. All statistical significance was determined using Student’s t-test with *p ≤ 0.05, **p ≤ 0.01, ***p ≤ 0.001, ****p ≤ 0.0001.

The loss of AR upon AKT inhibition (Fig. 4b) suggests that anti-androgens such as enzalutamide would be less effective when AKT is inhibited in ERG-positive cells. To test this, VCaP cells were treated with the GR_50_ of enzalutamide [25], with or without prior ipatasertib treatment. Enzalutamide and ipatasertib alone decreased VCaP cell number (Fig. 5a). However, enzalutamide did not further decrease cell number in ipatasertib treated cells. Surprisingly, enzalutamide increased cell numbers in cells with prior AKT inhibition (Fig. 5a). It is not clear why enzalutamide would have any effect in cells with low AR expression, but enzalutamide is known to exert effects through other nuclear hormone receptors such as glucocorticoid receptor [25].

Prostate cancer cells can respond to inhibition of AR signaling by becoming more mesenchymal [26, 27]. Our model (Fig. 4a) suggests that ERG-positive prostate cancer cells could adapt to loss of androgen signaling by reducing AKT signaling to promote EMT. To test this, we first characterized loss of androgen signaling in ERG-positive VCaP cells. VCaP cell proliferation decreased in phenol-red free, charcoal stripped media (Fig. 5b). The addition of the synthetic androgen R1881 (metribolone) rescued this decreased growth (Fig. 5b) suggesting that decreased growth was due to the loss of androgen signaling. To investigate how androgen depletion affects cell signaling pathways we compared cells grown in complete media to those grown in stripped media by immunoblotting. As expected, cells grown in stripped media had reduced AR as well as reduced ERG expression (*TMPRSS2::ERG* is expressed via the androgen-dependent *TMPRSS2* promoter) when compared to cells grown in complete media (Fig. 5c and d). Importantly, serum stripping reduced AKT phosphorylation without affecting total levels of AKT, consistent with our hypothesis that ERG-positive prostate cancer cells could adapt to loss of androgens by reducing AKT signaling. Interestingly, the amount of S215 phosphorylated ERG remained the same after charcoal stripping despite reduced levels of total ERG (Fig. 5c and d). ERG is phosphorylated downstream of MEK/ERK. We found that phosphorylated MEK (pMEK) significantly increased in the stripped VCaP cells when compared to cells grown in complete media suggesting increased activity of this pathway increases the ratio of phosphorylated ERG to total ERG (Fig. 5c and d). These data suggest that upon androgen depletion VCaP cells shift away from PI3K/AKT signaling and instead upregulate Ras/MAPK signaling allowing ERG to promote EMT.

### Both TLR4 and VEGFA inhibition decrease ERG-positive tumor growth.

**Figure 6:**
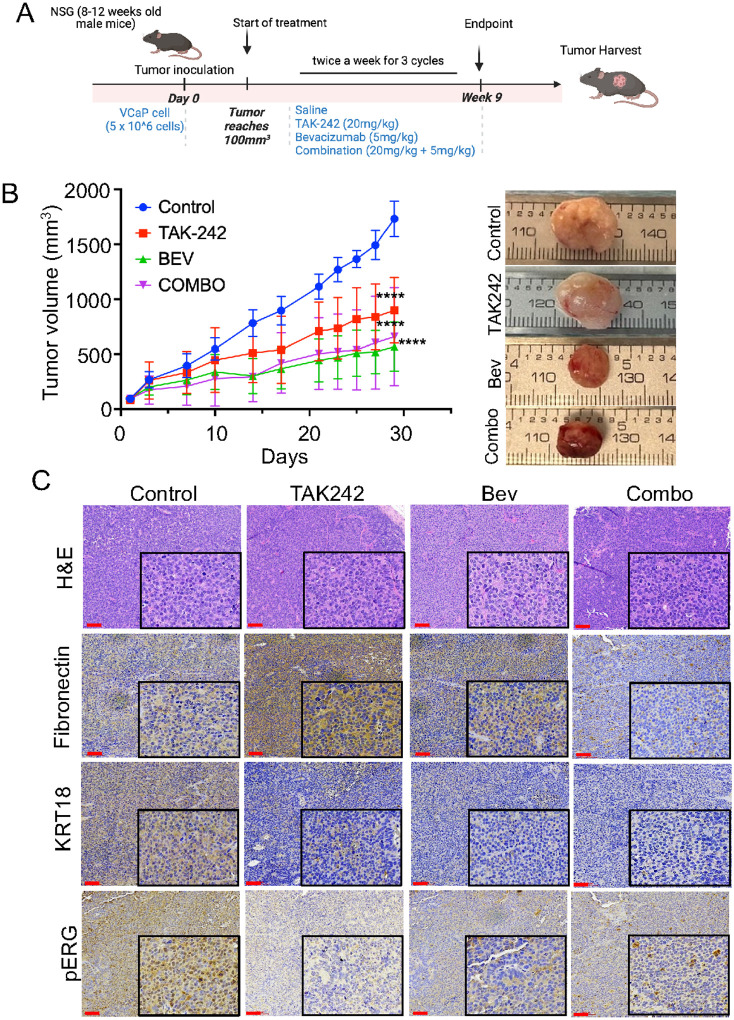
Inhibition of TLR4 and VEGFA decreases ERG-positive tumor growth. (**A**) Schematic representation of the mouse experiment (**B**) Mouse flank xenograft tumor growth was measured by caliper. A total of 3 × 10^6^ VCaP cells were injected. Tumors were allowed to grow to 100 mm^3^ and then treated with vehicle (DMSO), 20 mg/kg/day TAK-242, 5 mg/kg bevacizumab (Bev), or the combination. Mice received intraperitoneal injections twice per week for 3 weeks. Data presented as mean ± SEM. P values were derived from ANOVA, n=4, ****p < 0.0001. (**C**) Representative images of hematoxylin and eosin (H&E) staining of xenograft tumors and immunohistochemical staining for Fibronectin, KRT18, and S215 phosphorylated ERG (pERG). Images are shown at 40× magnification. Scale bar = 100 μm.

To evaluate the therapeutic impact of targeting ERG-associated signaling pathways in vivo, ERG-positive VCaP cells were grown as flank xenografts in male immunodeficient mice. Once tumors reached approximately 100 mm^3^, mice were treated with vehicle, the TLR4 inhibitor TAK-242 (20 mg/kg), the VEGFA inhibitor bevacizumab (5 mg/kg), or the combination of both agents (Fig. 6a). Both TAK-242 and bevacizumab treatment reduced tumor growth compared with vehicle controls (Fig. 6b). Combination treatment did not further reduce tumor growth relative to bevacizumab alone over the duration of the study (Fig. 6b). Despite similar caliper volume measurements, combination treated tumors had a distinct appearance compared to those treated with bevacizumab alone. The tumors from combination treated animals appeared more vascularized and less solid when dissected. Immunohistochemical analysis of xenograft tumors revealed treatment-dependent changes in tumor phenotype. Fibronectin and KRT18 staining demonstrated an increase in mesenchymal character and loss of luminal character for both single agent treatments (Fig. 6c). This would be consistent with both treatments switching the cell fate ERG is promoting towards mesenchymal. In contrast, combination-treated tumors showed loss of both luminal and mesenchymal markers suggesting loss of ERG function with regard to both fates (Fig. 6c). Levels of phosphorylated ERG were lowest in both bevacizumab and combination treatment groups, consistent with the smallest tumor size in these two groups. Together, these results indicate that while single agent treatments suppress tumor growth, they can promote a mesenchymal fate change, whereas combination treatment reduces tumor size without promoting a mesenchymal fate.

## DISCUSSION

Our findings support a model that ERG can promote distinct cell fates depending on the status of the PI3K/AKT signaling pathway. When AKT activity is high, ERG promotes luminal epithelial and luminal progenitor cell fates, and when AKT activity is low, ERG promotes mesenchymal and basal epithelial cell fates. We also find that ERG phosphorylation can be promoted by two different signaling pathways, the VEGF pathway and the TLR4 pathway. We find that the TLR4 pathway is important for ERG to promote mesenchymal and basal epithelial cell fates while the VEGF pathway is important for ERG to promote luminal epithelial cell fates. However, in some cases, such as in the VCaP xenograft tumor model, both pathways appear to function together to allow similar cell fate changes.

These findings have implications for the biology of ERG-positive castration resistant prostate cancer (CRPC). Recent studies indicate that there are several transcriptomic subtypes of CRPC based on the presence of AR, neuroendocrine markers, or the absence of both (double negative) [28, 29]. CRPC that is androgen signaling positive has luminal cell features, while double negative CRPC has mesenchymal and stem-like features [30]. Further, most CRPC tumors have a mixture of AR-positive and double negative cells present [30, 31]. Based on our findings, we propose that ERG could play a key role in driving both AR positive and double negative forms of CRPC and could promote conversion between these forms due to therapeutic pressure. Cellular plasticity often allows therapy resistance [32]. In particular, double negative (mesenchymal) CRPC becomes more prevalent when prostate cancer is treated with next-generation anti-androgens [26, 27]. Our findings suggest that decreases in PI3K/AKT signaling in ERG-positive tumors could promote double negative CRPC. Further, inhibition of TLR4 signaling could stop ERG from promoting this transition. Supporting this hypothesis, Han et al. lists *TLR4* as one of the top genes specifically up-regulated in double negative CRPC [30].

Our findings suggest that combining AR inhibition with inhibition of AKT signaling could be counter-productive within ERG-positive prostate cancer. This is because ERG promotes mesenchymal and basal epithelial cell fates in low-AKT backgrounds, and conversion to these cell types would allow tumor cells to become resistant to AR inhibition. However, our findings suggest other inhibitor combinations that could be effective in ERG-positive CRPC. One would be to combine TLR4 inhibition with VEGF inhibition. We found that this combination blocked the increased mesenchymal cell fates observed in the single-agent treatments. We did not observe a decrease in tumor size in the combination compared to single agents, but our findings warrant further studies that would extend these treatments to longer time points when resistance to the single agents might be more apparent. A second inhibitor combination suggested by our study would be an AR inhibitor plus a TLR4 inhibitor. An AR inhibitor such as enzalutamide would target AR-positive prostate cancer cells and the TLR4 inhibitor would stop ERG from promoting resistance by conversion to double negative (mesenchymal) disease. Finally, a similar strategy would be to stop ERG from promoting AR positive cell states by targeting AKT signaling while also stopping ERG from promoting mesenchymal states by targeting TLR4. All of these possible combinatorial approaches warrant further study for the potential to limit therapy resistance caused by ERG mediated cellular plasticity.

## Supplementary Files

This is a list of supplementary files associated with this preprint. Click to download.

- SupplementalFiles.pdf

## Data Availability

The datasets generated during the current study are available in the Gene Expression Omnibus (https://www.ncbi.nlm.nih.gov/geo/) under accession GSE330458.
